# Mtu1-Mediated Thiouridine Formation of Mitochondrial tRNAs Is Required for Mitochondrial Translation and Is Involved in Reversible Infantile Liver Injury

**DOI:** 10.1371/journal.pgen.1006355

**Published:** 2016-09-30

**Authors:** Yong Wu, Fan-Yan Wei, Layla Kawarada, Takeo Suzuki, Kimi Araki, Yoshihiro Komohara, Atsushi Fujimura, Taku Kaitsuka, Motohiro Takeya, Yuichi Oike, Tsutomu Suzuki, Kazuhito Tomizawa

**Affiliations:** 1 Department of Molecular Physiology, Faculty of Life Sciences, Kumamoto University, Kumamoto, Japan; 2 Precursory Research for Embryonic Science and Technology (PRESTO), Japan Science and Technology Agency (JST), Kawaguchi, Japan; 3 Department of Chemistry and Biotechnology, School of Engineering, The University of Tokyo, Tokyo, Japan; 4 Division of Developmental Genetics, Institute of Resource Development and Analysis, Kumamoto University, Kumamoto, Japan; 5 Department of Cell Pathology, Faculty of Life Sciences, Kumamoto University, Kumamoto, Japan; 6 Department of Molecular Genetics, Faculty of Life Sciences, Kumamoto University, Kumamoto, Japan; Max Planck Institute for Biology of Ageing, GERMANY

## Abstract

Reversible infantile liver failure (RILF) is a unique heritable liver disease characterized by acute liver failure followed by spontaneous recovery at an early stage of life. Genetic mutations in *MTU1* have been identified in RILF patients. MTU1 is a mitochondrial enzyme that catalyzes the 2-thiolation of 5-taurinomethyl-2-thiouridine (τm^5^s^2^U) found in the anticodon of a subset of mitochondrial tRNAs (mt-tRNAs). Although the genetic basis of RILF is clear, the molecular mechanism that drives the pathogenesis remains elusive. We here generated liver-specific knockout of Mtu1 (Mtu1^LKO^) mice, which exhibited symptoms of liver injury characterized by hepatic inflammation and elevated levels of plasma lactate and AST. Mechanistically, Mtu1 deficiency resulted in a loss of 2-thiolation in mt-tRNAs, which led to a marked impairment of mitochondrial translation. Consequently, Mtu1^LKO^ mice exhibited severe disruption of mitochondrial membrane integrity and a broad decrease in respiratory complex activities in the hepatocytes. Interestingly, mitochondrial dysfunction induced signaling pathways related to mitochondrial proliferation and the suppression of oxidative stress. The present study demonstrates that Mtu1-dependent 2-thiolation of mt-tRNA is indispensable for mitochondrial translation and that Mtu1 deficiency is a primary cause of RILF. In addition, Mtu1 deficiency is associated with multiple cytoprotective pathways that might prevent catastrophic liver failure and assist in the recovery from liver injury.

## Author Summary

Mitochondrial transfer tRNA (mt-tRNA) contains a variety of chemical modifications that are introduced post-transcriptionally. Three mt-tRNAs for Lys, Gln and Glu contain 5-taurinomethyl-2-thiouridine (τm^5^s^2^U) in their anticodons. It is known that the loss of 2-thiolation of τm^5^s^2^U is strongly associated with the development of reversible infantile liver failure (RILF) because pathogenic mutations of RILF were found in the *MTU1* gene, which encodes an enzyme responsible for the 2-thiolation of τm^5^s^2^U. However, the molecular mechanism underlying RILF pathogenesis associated with a lack of *MTU1* remains elusive. To understand the physiological function of MTU1 and its association with liver failure, we generated liver-specific Mtu1-deficient (Mtu1^LKO^) mice. Mtu1 deficiency abolished 2-thiouridine formation in the three mt-tRNAs. Loss of the 2-thiouridine modification resulted in a marked impairment of mitochondrial translation and abnormal mitochondrial structure. Consequently, the Mtu1^LKO^ mice exhibited liver injury, which resembles the symptoms of RILF patients. Furthermore, mitochondrial dysfunction in Mtu1^LKO^ mice induced mitochondrial biogenesis and suppressed oxidative stress. These findings elucidate the cellular and physiological functions of Mtu1 and provide a mouse model for understanding RILF pathogenesis.

## Introduction

Transfer RNA (tRNA) is an adaptor molecule that converts genetic information into an amino acid sequence in protein synthesis. tRNAs contain a wide variety of modified nucleosides that are introduced post-transcriptionally [1, 2]. In mammalian mitochondria, 22 subtypes of tRNAs encoded in mitochondrial DNA participate in the translation of 13 protein subunits of respiratory chain complexes in mitochondria. Fifteen species of modified nucleotides are found at 118 positions of bovine mitochondrial tRNAs (mt-tRNAs) [3]. A number of pathogenic point mutations associated with mitochondrial diseases are found in mt-tRNA genes [4–5]. Some of these mutations impair mt-tRNA modifications, leading to defective translation and mitochondrial dysfunction. In addition, a number of pathogenic mutations have been found in mt-tRNA-modifying enzymes, including *MTO1* [6–8], *GTPBP3* [9], *MTU1* [10–13], and *PUS1* [14], indicating that decreased modifications of mt-tRNAs caused by tRNA mutations and defective tRNA-modifying enzymes result in pathological consequences. Supporting these findings, the physiological roles of tRNA-modifying enzymes have been extensively studied in mouse models lacking mt-tRNA-modifying enzymes [15–16].

Unique to mitochondrial tRNA modifications, 5-taurinomethyluridine (τm^5^U) is present at the first position of the anticodon (i.e., the “wobble position” or position 34) of mt-tRNAs for Leu (UUR) and Trp, whereas its 2-thiouridine derivative (τm^5^s^2^U) is found at the same position of mt-tRNAs for Glu, Gln and Lys [17–18]. These modifications allow mt-tRNAs to recognize their cognate codons precisely and ensure accurate translation in the mitochondria. The enzymes mitochondrial tRNA translation optimization 1 (Mto1) and GTP binding protein 3 (Gtpbp3) are assumed to be responsible for τm^5^U formation [19]. In addition, mitochondrial tRNA-specific 2-thiouridylase 1 (MTU1) catalyzes the 2-thiolation of τm^5^U to form τm^5^s^2^U [19].

The lack of a yeast homolog of MTU1 resulted in impaired mitochondrial translation activity and a severe respiratory defect [19]. Moreover, acute knockdown of *MTU1* in HeLa cells reduced the oxygen consumption rate and resulted in a defective mitochondrial membrane potential [19]. Intriguingly, *MTU1* has been implicated in the pathogenesis of reversible infantile liver failure (RILF) [10–13], a life-threatening condition characterized by acute liver dysfunction during the first 2–4 months after birth. However, a majority of patients spontaneously recover and never exhibit another symptom [20]. Genomic analysis has identified a variety of autosomal recessive mutations, including substitutions, insertions and deletions, in the coding region of *MTU1* in RILF patients [20]. These mutations in *MTU1* are predicted to cause the loss of MTU1 activity, which subsequently triggers the pathological symptoms. Indeed, immortalized cell lines derived from RILF patients exhibit very low levels of MTU1 with a marked reduction of 2-thiolation levels in mt-tRNAs, leading to defective mitochondrial translation [21]. However, little reduction of mitochondrial translation has been observed in the fibroblasts of RILF patients [22], which raised some discrepancies by different groups regarding the observed MTU1 functions.

To reveal the physiological role of MTU1-mediated 2-thiouridine formation of τm^5^s^2^U in the regulation of mitochondrial protein translation in hepatocytes and its relevance in RILF, we generated multiple lines of conditional *Mtu1* knockout mice and investigated the molecular functions of Mtu1 in these murine models.

## Results

### Mtu1 is indispensable for embryonic development

**Fig 1:**
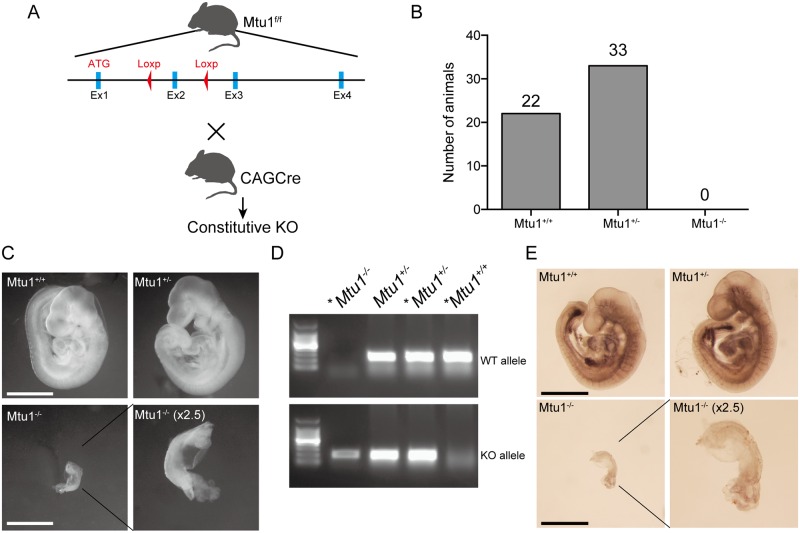
Mtu1 is essential for embryonic development. (A) Breeding strategy to generate constitutive Mtu1 knockout mice. ATG indicates the start codon. (B) Total number of wild-type (Mtu1^+/+^), heterozygous (Mtu1^+/-^) and homozygous (Mtu1^-/-^) animals obtained from parental Mtu1^+/-^ mice. (C) Morphology of Mtu1^+/+^, Mtu1^+/-^ and Mtu1^-/-^ embryos at stage E9. Bar = 5 mm. Note that the development of Mtu1^-/-^ embryos was severely delayed. (D) Genotyping analysis of embryos. DNA fragments corresponding to the wild-type (WT) allele and deleted (KO) allele are shown. Asterisks indicate the genotypes of the embryos shown in (C). (E) Blood vessel networks in embryos shown in (C) were examined by immunostaining against PECAM-1.

To investigate the physiological role of Mtu1, we generated constitutive Mtu1 knockout mice (Mtu1^-/-^) (Fig 1A). However, no Mtu1^-/-^ mice were obtained after multiple generations of breeding (Fig 1B). The embryos at 9 days post-coitum (E9) were isolated for morphological examination. While the morphology of Mtu1^+/-^ embryos did not differ from that of Mtu1^+/+^ embryos, the size of Mtu1^-/-^ embryos was strikingly small (Fig 1C and 1D). To visualize the internal structure, the embryos were stained with platelet endothelial cell adhesion molecule-1 (PECAM-1). Mtu1^+/+^ and Mtu1^+/-^ embryos exhibited organized blood vessel networks that surrounded well-developing tissues, such as the heart, brain and spinal cord (Fig 1E). In contrast, neither blood vessel networks nor developmental stage-matched tissues were observed in Mtu1^-/-^ embryos (Fig 1E). Considering these morphologies, we concluded that the Mtu1^-/-^ embryo died at a developmental stage as early as E7.5~8.

### Liver-specific Mtu1 knockout mice exhibited liver injury and altered metabolism

**Fig 2:**
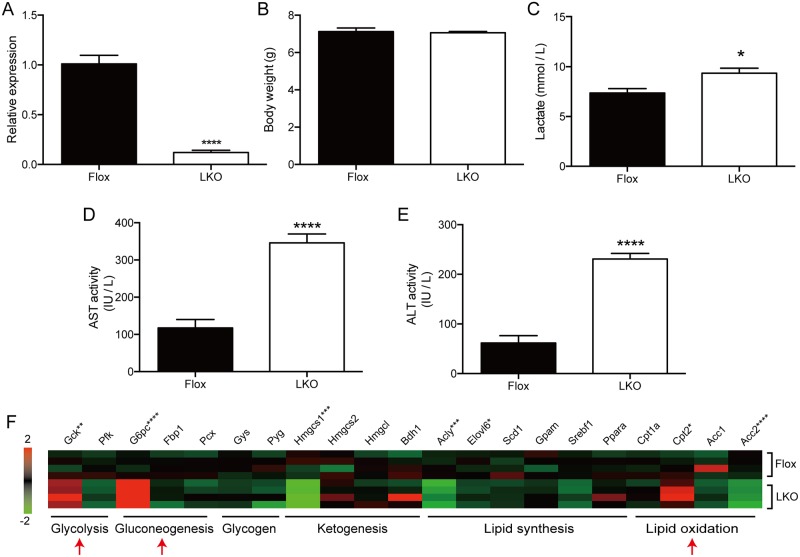
Hepatocyte-specific Mtu1 knockout mice exhibited liver injury and altered metabolism. (A) Relative expression levels of *Mtu1* in liver tissues of control (Flox) and Mtu1^LKO^ (LKO) mice. n = 3 each. ****P<0.0001. (B) The body weight of male mice at 3 weeks of age. n = 3 each. (C-E) Plasma lactate (C), aspartate aminotransferase (AST) (D) and alanine aminotransferase (ALT) (E) of Mtu1^LKO^ and control male mice at 3 weeks of age. n = 6 each. *P < 0.05, ****P < 0.0001. (F) Expression levels of selected metabolism-related genes in the livers of Mtu1^LKO^ and control male mice at 3 weeks of age were examined by quantitative PCR (n = 4 each). The relative expression levels are shown as a heat map. *P <0.05, **P < 0.01, ***P < 0.001, ****P < 0.0001.

To avoid the embryonic lethality, we generated liver-specific Mtu1 knockout mice (Mtu1^LKO^) (S1 Fig). The Mtu1^LKO^ mice were viable and developed without any obvious morphological defects (S1 Fig). We confirmed a 10-fold reduction in the *Mtu1* transcript levels in the liver of Mtu1^LKO^ mice compared to Mtu1^Flox^ mice (Fig 2A). The average body weight of Mtu1^LKO^ mice did not differ from that of Mtu1^Flox^ mice (Fig 2B). Laboratory examinations of 3-week-old mice revealed significantly elevated plasma levels of lactate, aspartate aminotransferase (AST) and alanine aminotransferase (ALT) in Mtu1^LKO^ mice (Fig 2C–2E). In addition, the Mtu1^LKO^ mice exhibited a high level of serum lactate dehydrogenase (LDH) and a low level of albumin (ALB) compared to the Mtu1^Flox^ mice (Table 1). These results clearly indicate that liver injury occurs in the Mtu1^LKO^ mice. Interestingly, the Mtu1^LKO^ mice exhibited altered metabolism. The levels of total cholesterol (T-CHO), high-density lipoprotein cholesterol (HDL-C), amylase (AMY), creatinine (CRE) and calcium (Ca) were significantly reduced in the Mtu1^LKO^ mice (Table 1). In agreement with these biochemical results, a gene expression analysis revealed an increase in the expression levels of genes involved in glycolysis (*Glucokinase*, *Gck*), gluconeogenesis (*Glucose-6-phosphatase catalytic subunit*, *G6pc*), and lipid oxidation (*carnitine palmitoyltransferase 2*, *Cpt2*) in the Mtu1^LKO^ mice (Fig 2F).

**Table 1:** Serum biochemical data of Mtu1^Flox^ and Mtu1^LKO^ mice.

	Flox	LKO	*P* Value
**LDH (IU / L)**	**693±120.488**	**1117±90.376**	**0.01830**
CK (IU / L)	1312.667±394.316	760±76.969	0.19922
**AMY (IU / L)**	**2177.667±53.922**	**1799.5±19.711**	**0.00006**
**T-CHO (mg / dL)**	**88.333±5.207**	**74.167±2.056**	**0.02984**
TG (mg / dL)	19.167±1.662	20.833±1.778	0.50899
**HDL-C (mg / dL)**	**58.133±3.285**	**48.15±2.224**	**0.03058**
LDL-C (mg / dL)	8.157±0.654	9.667±0.919	0.21310
TP (g / dL)	4.657±0.058	4.188±0.261	0.11051
**ALB (g / dL)**	**0.718±0.031**	**0.602±0.037**	**0.03761**
UN (mg / dL)	37.95±7.281	24.15±0.595	0.08818
UA (mg / dL)	2.333±0.15	2.15±0.076	0.30118
**CRE (mg / dL)**	**0.313±0.067**	**0.132±0.006**	**0.02277**
**Ca (mg / dL)**	**8.133±0.249**	**7.2±0.301**	**0.03793**
IP (mg / dL)	11.217±1.227	10.333±0.383	0.50747
GLU (mg / dL)	207.833±22.886	189.933±11.268	0.49653

Abbreviations are as follows: LDH, lactate dehydrogenase; CK, creatine kinase; AMY, amylase; T-CHO, total cholesterol; TG, triglycerides; HDL-C, high-density lipoprotein cholesterol; LDL-C, low-density lipoprotein cholesterol; TP, total protein; ALB, albumin; UN, urea nitrogen; UA, uric acid; CRE, creatinine; Ca, calcium; IP, inorganic phosphorus; and GLU, glucose. n = 6. Bold numbers indicate statistical significance.

**Fig 3:**
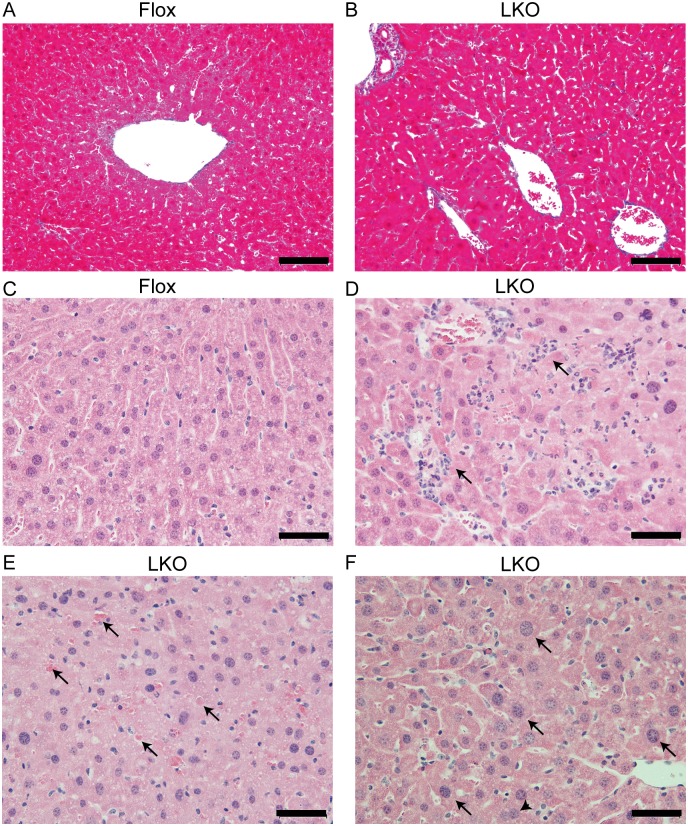
Hepatocyte-specific Mtu1 knockout mice exhibited liver injury. (A-B) Masson trichrome staining of liver sections from Flox and LKO mice. Bars = 0.2 mm. (C-F) High magnification views of representative H&E staining of liver sections from Flox (C) and LKO (D-F) mice. Bars in panels v-viii = 0.05 mm. (D) Representative macrophage infiltration (arrows) in an LKO mouse liver section. (E) Representative spotty necrosis (arrows) in an LKO mouse liver section. (F) Representative hepatocytes with karyomegaly (arrows) and multiple nuclei (arrowhead) in an LKO mouse liver section.

We next investigated liver injury in the Mtu1^LKO^ mice at the histological level. The Mtu1^LKO^ mice exhibited normal liver structures compared to Mtu1^Flox^ mice (S1 Fig). No obvious fibrosis was observed in the livers of Mtu1^LKO^ mice by Masson trichrome staining (Fig 3A and 3B). Interestingly, there was an infiltration of macrophages (arrows in Fig 3C and 3D) and spotty necrosis (arrows in Fig 3E) in the livers of Mtu1^LKO^ mice. In addition, enlarged hepatocytes with karyomegaly and multiple nuclei were observed in the Mtu1^LKO^ mice (arrows in Fig 3F). These histological features of Mtu1^LKO^ mice resemble the clinical features of RILF patients [20].

### Mtu1-mediated s^2^ modification is essential for mitochondrial translation

**Fig 4:**
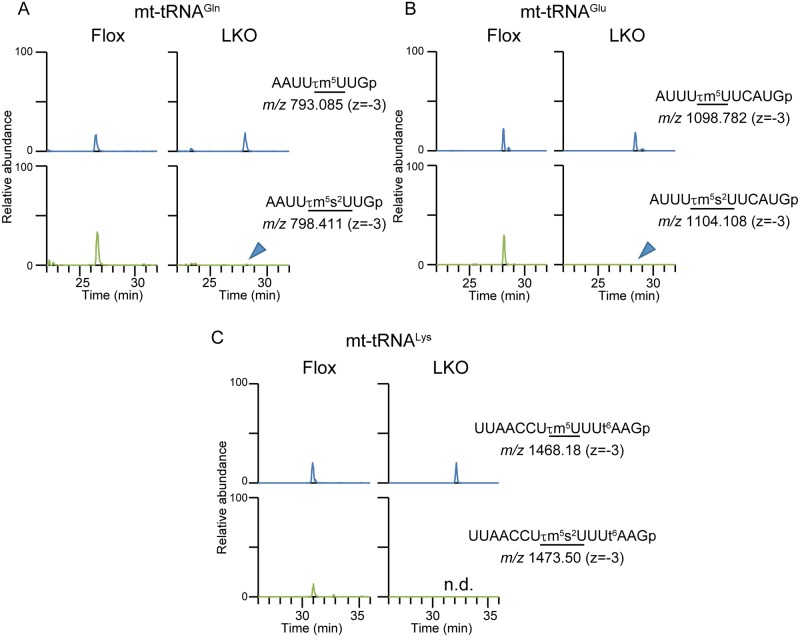
Loss of s^2^ modification in Mtu1-deficient liver. (A-C) Representative mass chromatograms of mt-tRNA^Gln^ (A), mt-tRNA^Glu^ (B) and mt-tRNA^Lys^ (C) purified from the livers of control (Flox) and knockout (LKO) mice. Arrowheads indicate traces of fragments containing s^2^ modification in LKO mice. Note that the levels of s^2^-containing mt-tRNAGln, mt-tRNAGlu and mt-tRNALys were almost absent in LKO mice. n.d.: not detected. n.d.: not detected.

To clarify the molecular function of Mtu1 and its association with liver injury, we examined mt-tRNA modifications and mitochondrial translation in Mtu1-deficient hepatocytes. mt-tRNA^Gln^, mt-tRNA^Glu^ and mt-tRNA^Lys^ were individually purified from liver tissues and subjected to mass spectrometry analysis. Interestingly, these mt-tRNAs were partially s^2^-modified even in the livers of Mtu1^Flox^ mice; 40~70% of the mt-tRNA^Glu^, mt-tRNA^Gln^ and mt-tRNA^Lys^ contained τm^5^s^2^U and s^2^U modifications, whereas the remaining 30~60% of the mt-tRNA contained τm^5^U modifications and unmodified U (S2 Fig). In the livers of Mtu1^LKO^ mice, the s^2^ modification was nearly absent in the three mt-tRNAs (Fig 4A–4C). There was a trace of τm^5^s^2^-containing mt-tRNAs, but this result was most likely derived from non-hepatic cells. Notably, the level of τm^5^U in mt-tRNA^Glu^, mt-tRNA^Gln^ and mt-tRNA^Lys^ remained unchanged despite of the loss of the s^2^ modification (S2 Fig). In addition to mitochondrial s^2^ modification, we examined other sulfur-containing modifications, including mcm^5^s^2^U (5-methoxycarbonylmethyl-2-thiouridine) modification in cytosolic tRNAs and ms^2^i^6^A (2-methylthio-N6-isopentenyladenosine) modification in mt-tRNAs. Interestingly, the abundance of these modifications was up-regulated in the livers of Mtu1^KO^ mice compared to Mtu1^Flox^ mice (S3 Fig).

**Fig 5:**
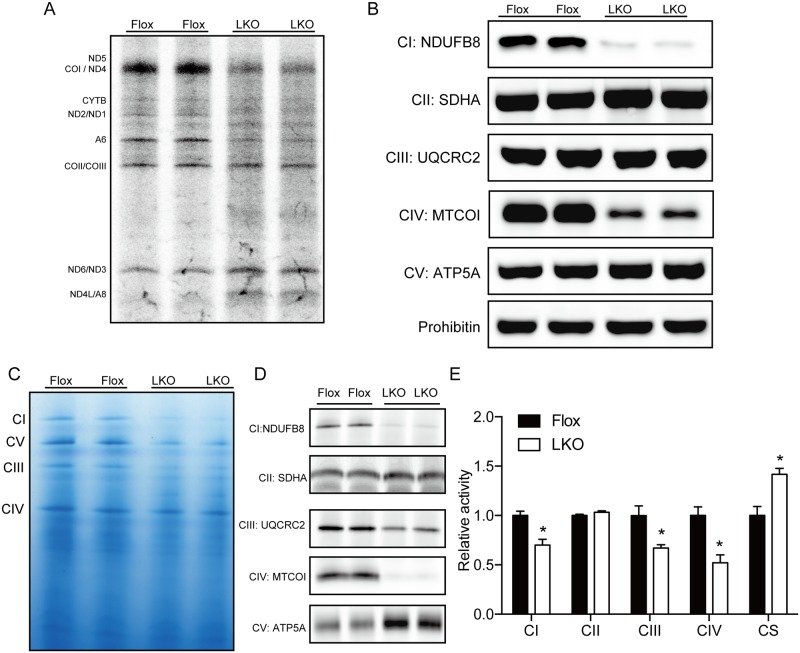
Mtu1 deficiency impaired mitochondrial translation and respiratory activities in hepatocytes. (A) Autoradiogram of mitochondrial translation in primary hepatocytes isolated from control (Flox) and hepatocyte-specific Mtu1 knockout (LKO) mice. (B) Protein levels of representative mitochondrial proteins were examined by western blotting. CI~CV indicates Complexes I ~ V. (C) Representative blue-native gel image of respiratory complexes purified from Flox and LKO mice. (D) Mitochondrial proteins incorporated in Complexes I ~ V were examined by blue-native PAGE followed by western blotting. (E) The activities of Complexes I ~ IV in the livers of Flox and LKO mice are shown. Individual complex activity was normalized to the activity of citrate synthase (CS). n = 3 each. **P < 0.01, ***P < 0.001.

Next, we examined mitochondrial protein translation in primary hepatocytes isolated from Mtu1^LKO^ and Mtu1^Flox^ mice. The level of mitochondrial translation was markedly reduced in Mtu1-deficient hepatocytes compared with control cells (Fig 5A). Interestingly, the degree of impairment in 13 mitochondrial proteins depended on their molecular weights. The translation of 9 mitochondrial proteins with molecular weights higher than 25 kDa (equal to the molecular weight of COII/III) was markedly impaired in Mtu1-deficient cells (Fig 5A). On the other hand, the translation of the remaining 4 mitochondrial proteins (ND6, ND3, ND4L and A8) with molecular weights lower than 25 kDa was only slightly increased in Mtu1-deficient cells (Fig 5A).

The impairment in mitochondrial translation resulted in a decrease in the steady-state level of mitochondrial proteins (Fig 5B). Notably, the protein levels of NDUFB8 and MTCOI, which comprise Complexes I and IV, respectively, showed a marked reduction in the livers of Mtu1^LKO^ mice (Fig 5B). Accordingly, the formation of respiratory Complexes I~IV was impaired in Mtu1^LKO^ mice (Fig 5C and 5D). The disruption of mitochondrial translation consequently resulted in a broad and significant decrease in the activities of Complexes I, III and IV (Complex I: 70%, Complex III: 67%, Complex IV: 52% versus Flox mice, Fig 5E). There was also a significant increase of citrate synthase activity in the livers of Mtu1^LKO^ mice. These results clearly demonstrate that Mtu1 is indispensable for mitochondrial translation and respiratory activities.

### Aberrant mitochondrial morphology in Mtu1-deficient hepatocytes

**Fig 6:**
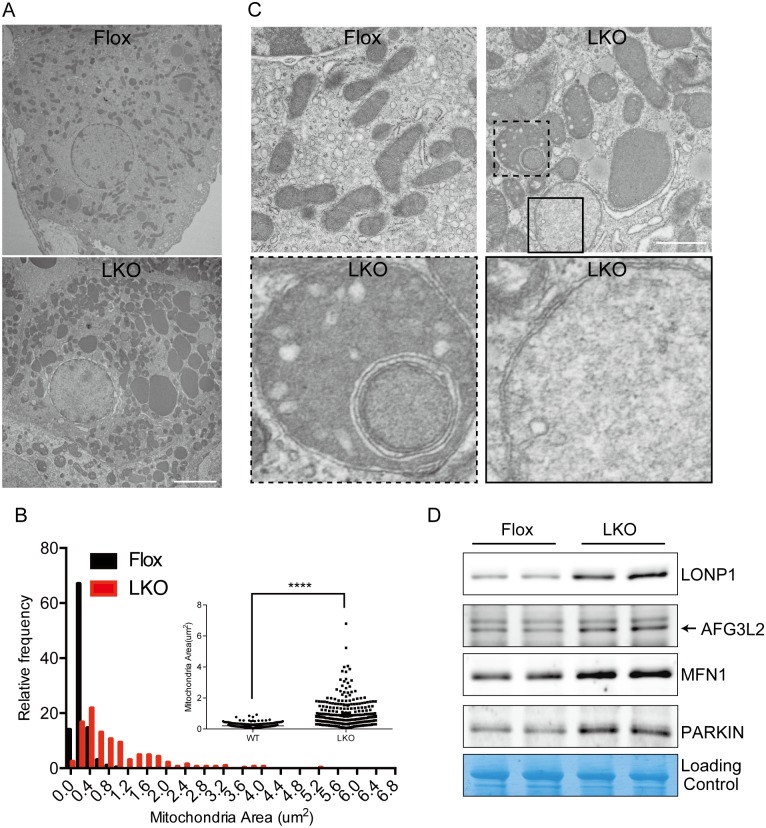
Aberrant mitochondrial morphology in Mtu1-deficient hepatocytes. (A) Representative electron microscopy images of hepatocytes in control (Flox) and hepatocyte-specific Mtu1 knockout (LKO) mice. Bar = 5 μm. (B) The mitochondrial area in Flox and LKO hepatocytes was examined by histogram and scatter plot (Insert). n = 300 and 330 for Flox and LKO, respectively. ***P < 0.001. (C) Representative images of hepatic mitochondria at high magnification. Bar = 1 μm. Boxes with dashed and solid lines indicate aberrant structures magnified in lower panels. (D) Examination of the amounts of proteins related to mitochondrial proteostasis by western blotting. Total mitochondrial proteins were stained and used as a loading control. Arrow indicates bands corresponding to AFG3L2.

Because mitochondrial dynamics are closely coupled with mitochondrial proteostasis [23], we examined mitochondrial morphology using electron microscopy. Striking mitochondrial enlargement and proliferation were observed in Mtu1-deficient hepatocytes (Fig 6A). The average mitochondrial area in Mtu1-deficient hepatocytes was 4.3-fold larger than that in control hepatocytes (Fig 6B). Notably, nearly all mitochondria in Mtu1-deficient hepatocytes exhibited aberrant cristae structures. The cristae were either abnormally swollen or lost in most of the mitochondria (Fig 6C). Some mitochondria contained an inner vacuole with multiple layers of membrane, and some exhibited very low electron density (Fig 6C). Disruption of mitochondrial function has been associated with the activation of the pathway related to mitochondrial proteostasis [24]. In agreement with the previous findings, there was a substantial increase in proteins related to proteostasis, such as LONP1, AFG3L2, DRP1, MFN1 and PARKIN (Fig 6D). Taken together, these results demonstrate that Mtu1-mediated mitochondrial translation is required for the maintenance of mitochondrial structures and proteostasis.

### Activation of compensatory signaling in Mtu1-deficient hepatocytes

**Fig 7:**
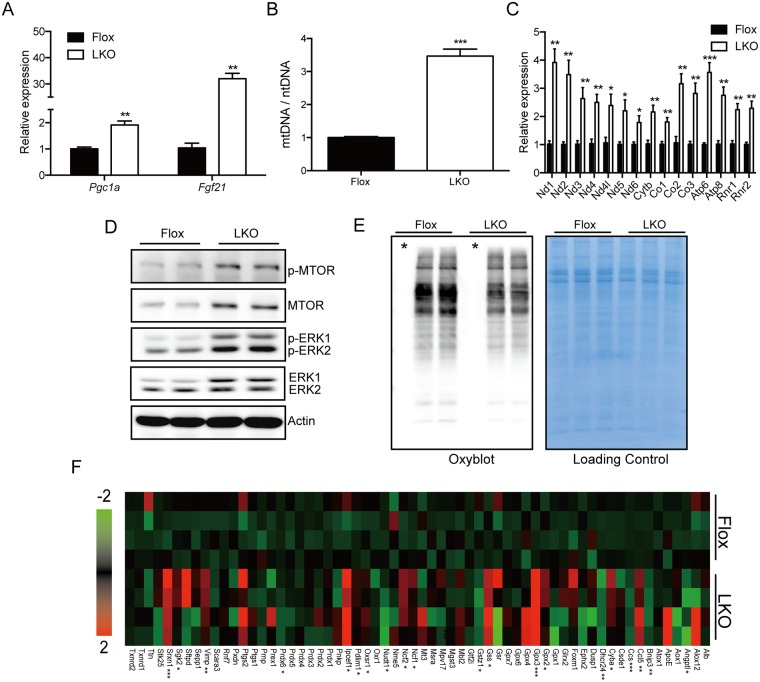
Enhancement of mitochondrial biogenesis and oxidative stress response in Mtu1-deficient hepatocytes. (A) Relative expression of *Pgc1α* and *Fgf21* in the livers of control (Flox) and knockout (LKO) mice. n = 4 each. **P < 0.01. (B) The ratios of mitochondrial DNA (mtDNA) levels to nuclear DNA (nDNA) levels in the livers of Flox and LKO mice are shown. n = 4 each. ***P < 0.001. (C) Relative expression of mitochondrial DNA-encoded mitochondrial genes in the livers of Flox and LKO mice. n = 4 each. *P < 0.05, **P < 0.01. (D) Western blotting revealing an increase in MTOR, phospho-MTOR (p-MTOR), ERK1/2 and phospho-ERK1/2 levels in primary hepatocytes. (E) Examination of protein carbonylation by Oxyblot in mitochondria isolated from Flox and LKO livers. Total mitochondrial proteins were stained with CBB and used as a loading control. Asterisks indicate negative control samples. (F) Expression levels of genes related to the oxidative stress response were examined by quantitative PCR (n = 4 each). The relative expression levels are shown in a heat map. *P <0.05, **P < 0.01, ***P < 0.001.

The Mtu1^LKO^ mice showed severe mitochondrial dysfunction in hepatocytes but maintained liver function. We investigated the molecular mechanism that enables Mtu1^LKO^ mice to tolerate severe mitochondrial dysfunction in hepatocytes. Mitochondrial dysfunction often induces an increase in hepatic Fgf21 levels, which has been associated with compensatory signaling, such as mitochondrial biogenesis [25, 26]. There was an approximately 32-fold increase in the *Fgf21* levels in the livers of Mtu1^LKO^ mice (Fig 7A). Accordingly, peroxisome proliferator-activated receptor gamma coactivator 1-alpha (*Pgc1α*), an upstream regulator of mitochondrial proliferation, was significantly up-regulated (Fig 7A). Accordingly, there was an approximately 3-fold increase of the mtDNA copy number, which was associated with an approximately 4-fold increase in the mtDNA-encoded mitochondrial genes in Mtu1-deficient hepatocytes (Fig 7B–7C). In addition, MTOR and ERK1/2, which are effectors of FGF21 [27–28], were also up-regulated at both the total protein level and phosphorylation level in Mtu1-deficient primary hepatocytes (Fig 7D).

Mitochondria are the major source for the production of reactive oxygen species, and its dysfunction has been associated with oxidative stress [29]. We examined how oxidative stress was managed in Mtu1-deficient hepatocytes. Surprisingly, there was a decrease in mitochondrial protein carbonylation, which is a byproduct of oxidative stress, in the livers of Mtu1^LKO^ mice (Fig 7E). In addition, we examined the levels of oxidative stress-related metabolites in the liver tissues (S4 Fig). The glutathione (GSH) and cysteine levels in Mtu1^LKO^ mice did not differ from those in Mtu1^Flox^ mice. Interestingly, the level of glutathione disulfide (GSSG), a marker of oxidative stress, trended toward a decrease in the livers of Mtu1^LKO^ mice (S4 Fig). These results prompted us to examine the gene profiles that are related to oxidative stress in liver tissues. These genes were dynamically changed in the livers of Mtu1^LKO^ mice, indicating an active response to oxidative stress in the Mtu1^LKO^ mice (Fig 7F). Importantly, antioxidant genes, such as glutathione synthetase (Gss), glutathione peroxidase (Gpx2/3) and sulfiredoxin 1 (Srxn1), were significantly up-regulated. Together, these results show that the increase in mitochondrial biogenesis in combination with the adaptive scavenging of oxidative stress might compensate for the mitochondrial dysfunction and prevent catastrophic liver failure in Mtu1^LKO^ mice.

### Sustained liver function and compensatory signaling in adult Mtu1^LKO^ mice

**Fig 8:**
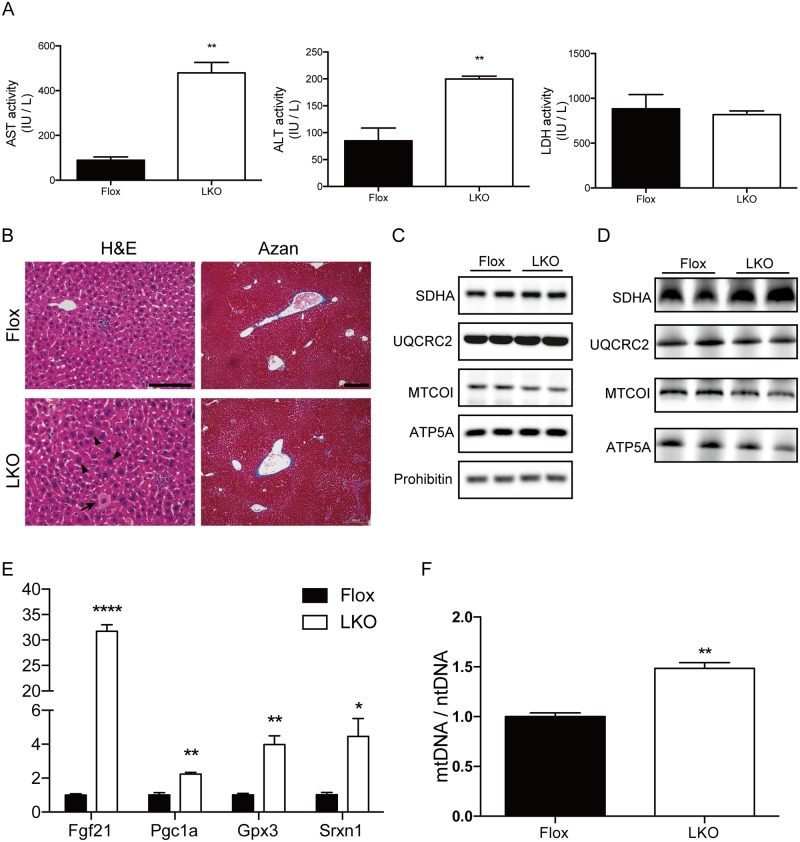
Sustained liver function in adult Mtu1-deficient mice. (A) Serum AST, ALT and LDH levels in LKO and Flox mice at the age of 16 weeks. n = 3 per genotype, **P < 0.01. (B) H&E and Azan staining in 16-week-old LKO and Flox mice. The arrow indicates spotty necrosis. Arrowheads indicate enlarged hepatocytes with karyomegaly. Bar = 0.2 mm for H&E staining. Bar = 0.1 mm for Azan staining. (C) Protein levels of representative mitochondrial proteins in the livers of 16-week-old Flox and LKO mice were examined by western blotting. (D) Mitochondrial proteins incorporated in Complexes I ~ V were examined by blue-native PAGE followed by western blotting. (E) The relative expression levels of *Fgf21*, *Pgc1a*, *Gpx3* and *Srxn1* in the livers of Flox and LKO mice are shown. n = 3 each. *P < 0.05, **P < 0.01, ****P < 0.0001. (F) The ratios of mtDNA levels to ntDNA levels in the livers of 16-week-old Flox and LKO mice are shown. n = 3 each. **P < 0.01.

Finally, to evaluate the progression of liver injury in Mtu1^LKO^ mice, we examined the histological, biochemical and genetic features of the livers at 16 weeks. The Mtu1^LKO^ mice were alive and exhibited sustained liver function. Serum ALT and AST levels in 16-week-old Mtu1^LKO^ mice, which were at the same levels as those of 3-week-old Mtu1^LKO^ mice, were still higher than the levels in 16-week-old Mtu1^Flox^ mice (Fig 8A). Unlike the 3-week-old mice, the serum LDH levels in the 16-week-old Mtu1^LKO^ mice did not differ from those of the Mtu1^Flox^ mice (Fig 8A). Similar to the histological features of 3-week-old mice, 16-week-old Mtu1^LKO^ mice also exhibited enlarged hepatocytes with karyomegaly and spotty necrosis; however, no obvious fibrosis was observed (Fig 8B).

Biochemical examination of mtDNA-derived MTCOI in total tissue lysates and in purified respiratory complexes revealed that the protein levels of MTCOI remained at low levels in 16-week-old Mtu1^LKO^ mice (Fig 8C–8D). Nevertheless, it is worthwhile to indicate that MTCOI levels appeared to be slightly increased compared to 3-week-old Mtu1^LKO^ mice (Fig 5D). At the gene expression level, Mtu1^LKO^ mice exhibited a marked up-regulation of *Fgf21* at 16 weeks (Fig 8E). Genes related to mitochondrial proliferation and suppression of oxidative stress, such as *Pgc1a*, *Gpx3* and *Srxn1*, also remained up-regulated in Mtu1^LKO^ mice compared to age-matched Mtu1^flox^ mice (Fig 8E). Accordingly, the relative mt-DNA copy number was up-regulated in 16-week-old Mtu1^LKO^ mice (Fig 8F). Taken together, these results suggest that despite the lasting mitochondrial dysfunction from embryo to adulthood, Mtu1^LKO^ mice were able to adapt to the liver injury and even exhibited some features of recovery in adulthood.

## Discussion

In the present study, we established hepatocyte-specific Mtu1-deficient mice that manifest the clinical symptoms of RILF. Mtu1 deficiency in hepatocytes resulted in a marked reduction in mitochondrial translation. The impairment in mitochondrial translation subsequently caused a broad decrease in respiratory activities and led to the disruption of membrane integrity. The mitochondrial dysfunction consequently induced liver injury. Our murine model provides mechanistic insights into the pathogenesis of RILF.

Mutations in *TRMU (MTU1)* have been implicated in the pathogenesis of RILF [20]. To date, 20 RILF patients carrying pathogenic mutations in the coding region of *MTU1* have been reported [20]. In contrast to the genetic evidence, the mechanism of RILF remains controversial. Some studies have reported that Mtu1 deficiency alone does not always cause the decrease in mitochondrial translation in RILF patient-derived cell lines [21–22]. In contrast, we showed that Mtu1 deficiency resulted in a marked impairment in mitochondrial translation and respiration in yeast and human cancer cell lines [19]. This discrepancy was likely caused by different experimental conditions in these studies. Indeed, all these studies were performed in cells that originated from different species. Notably, none of the previous studies were performed in cells related to the liver, which is the symptomatic tissue in RILF. In the present study, we generated liver-specific Mtu1 knockout mice, which allow us to investigate the role of Mtu1 in hepatocytes for the first time. Consistent with our previous results, Mtu1 deficiency markedly inhibited mitochondrial translation in hepatocytes, resulting in a substantial decrease in respiratory complexes and activities. Thus, our results lead to a clear conclusion that Mtu1 is required for mitochondrial translation in hepatocytes.

Why is Mtu1-mediated s^2^ modification crucial for mitochondrial translation? A previous study showed that bacterial tRNA^Gln^ lacking the s^2^ modification was still capable of translating the GAA codon, but the decoding efficiency of s^2^-deficient tRNA^Gln^ was 4-fold lower than that of a fully modified tRNA^Gln^ [30]. Therefore, it is conceivable that the major role of the s^2^ modification at 34U is to facilitate base-pairing with A to increase decoding efficiency. In mice and humans, mitochondrial genes predominantly utilize the GAA, CAA and AAA codons, which are decoded by mt-tRNA^Glu^, mt-tRNA^Gln^ and mt-tRNA^Lys^, respectively (S5 Fig). It is likely that mitochondria perverted the s^2^ modification during evolution for the optimal translation of biased codons. In addition, the codon usage might also influence the translation efficiency. In fact, the abundance of GAA, CAA and AAA codons is proportional to the length of mRNA, whereas the frequency of these codons did not differ among the mitochondrial genes (S5 Fig). Therefore, s^2^ modification would be particularly required for the efficient translation of long mRNAs because of their high demand for their corresponding tRNAs.

Despite the important role of s^2^ modification in regulating efficient translation, the s^2^ modification level only ranges from 40%~70% at steady state. It is likely that GAA, CAA and AAA codons would be preferentially and efficiently decoded by fully modified mt-tRNAs in wild-type mitochondria. However, the unmodified mt-tRNAs might still be partially functional because mitochondrial translation was still detectable in Mtu1-deficient cells. Supporting this speculation, a complete loss of the mcm^5^s^2^ modification, the cytosolic counterpart of τm^5^s^2^, in a subset of yeast cytosolic tRNAs resulted in a lethal phenotype that was rescued by overexpressing unmodified tRNA^Lys(UUU)^ [31].

The Mtu1^LKO^ mice showed hepatic inflammation, necrosis, and elevated plasma levels of lactate and AST. These pathological features closely mimic the clinical symptoms of RILF [20]. However, compared with RILF patients, the phenotypes of Mtu1^LKO^ mice were rather moderate. Notably, the Mtu1^LKO^ mice did not exhibit hepatic fibrosis and nodule formation, which are frequently observed in RILF patients [20]. The genetic background of the Mtu1^LKO^ mice might explain the mild hepatic injury. There is substantial evidence to suggest that mice with the C57BL6 background are resistant to hepatic fibrosis [32–33]. Despite this resistance, the Mtu1^LKO^ mice still exhibited symptoms of liver injury, which further emphasizes the critical role of Mtu1-mediated mitochondrial translation in the development of RILF.

While the hepatocyte-specific Mtu1 knockout mice were viable, the constitutive knockout mice were embryonic lethal at a very early developmental stage. In agreement with our results, the constitutive deficiency of murine Dars2 (mitochondrial aspartyl-tRNA synthetase), which is a component of the mitochondrial translation machinery, is also embryonic lethal at a very early developmental stage [26]. These results suggest that efficient mitochondrial translation is indispensable for embryonic development. Importantly, lethality due to Mtu1 deficiency has been observed in RILF patients. Indeed, 6 of 20 patients died of acute liver failure between 1 and 8 months of age [20]. Three of the 6 patients carried homozygous mutations in either the translational start codon (Met1Lys) or the active site (Asn96Ser). These mutations are predicted to cause a complete loss of Mtu1 or its enzymatic activity in the patients. In contrast, many of the surviving patients carried mutations in non-essential domains, which presumably cause a partial inhibition of Mtu1 activity. Taken together, these results suggest that the clinical progression of RILF might depend on the level of remaining Mtu1 activity in the tissues.

Mtu1-mediated s^2^ modification requires a complicated enzymatic reaction that transfers the sulfur atom from cysteine to tRNAs [34]. Because cysteine metabolism is limited during neonatal development, it is proposed that cysteine availability might contribute to the development of liver failure in patients carrying pathogenic mutations [11, 21] However, the cysteine levels in liver tissues of Mtu1^LKO^ mice did not differ from those of Mtu1^Flox^ mice. In addition, sulfur-containing tRNA modifications, including cytosolic mcm^5^s^2^U modifications and mitochondrial ms^2^i^6^A modifications, remained intact in Mtu1-deficient cells. Our results suggest that cysteine availability is likely not involved in the pathological phenotypes in our mouse model.

The reversibility is the most interesting feature of RILF. After surviving the acute phase, the patients spontaneously recover without recurrence due to unknown mechanisms [20]. Mitochondrial biogenesis is a potential compensatory effect for mitochondrial dysfunction [26]. Indeed, we observed marked mitochondrial biogenesis in response to Mtu1 deficiency. Mitochondrial biogenesis is most likely activated by up-regulation of *Pgc1α* signaling. This result was consistent with the observation in Dars2 knockout mice, which also showed up-regulation of *Pgc1α* expression and mitochondrial biogenesis [26]. In addition to the mitochondrial effect, our study revealed a unique compensatory effect involving suppression of oxidative stress. Although mitochondrial dysfunction is usually associated with the generation of oxidative stress [29], there was a moderate reduction in the stress levels and a marked increase in antioxidant gene expression in Mtu1-deficient hepatocytes. It is likely that the Mtu1 deficiency triggers oxidative stress due to severe mitochondrial dysfunction, but the adaptive scavenging response is strong enough to suppress the stress to a rather low level. Intriguingly, up-regulation of antioxidant genes in Mtu1^LKO^ mice has been observed from the adolescent stage to the adult stage. The continuous activation of mitochondrial biogenesis and the suppression of oxidative stress might protect Mtu1^LKO^ mice from catastrophic liver failure and maintain liver function in a tolerable condition from embryo to adulthood. From this perspective, treating RIFL patients with either cysteine or N-acetylcysteine might suppress oxidative stress and assist an early recovery.

In addition to RILF, the Mtu1-mediated s^2^ modification has also been implicated in reversible infantile respiratory chain deficiency (RIRCD) [20]. RIRCD patients exhibit severe myopathy in the first months of life, followed by spontaneous recovery with some mild residual myopathy. The molecular mechanism underlying RIRCD is unknown; however, genetic analysis has revealed a single homoplasmic m.14674T>C mutation in mitochondrial DNA that corresponds to mt-tRNA^Glu^ [35–37]. Interestingly, there was a decrease in the s^2^ modification in mt-tRNA^Glu^ in muscle samples with an m.14674T>C mutation [21]. Similar to RILF, Boczonadi et al. reported that the decrease in s^2^ modification was not associated with the impairment of mitochondrial translation in fibroblasts and myoblast cells established from RIRCD patients [21]. Given the different regulatory mechanisms in immortal cells and intact tissues, it is likely that the m.14674T>C mutation might also affect mitochondrial translation in the tissues of RIRCD patients. Further study using muscle-specific Mtu1-deificient mice may shed light on the molecular mechanism of RIRCD.

In summary, we demonstrated that Mtu1-mediated s^2^ modification of mt-tRNA is indispensable for efficient mitochondrial translation and activities. Our study suggests that mitochondrial dysfunction due to Mtu1 deficiency is the primary cause of RILF. Our murine model is a valuable tool for understanding the molecular mechanism of RILF and for developing effective treatments.

## Materials and Methods

### Animals

Constitutive Mtu1 knockout mice were generated by crossing transgenic mice harboring exon 2 of the *Mtu1* gene floxed by LoxP sequences (Mtu1^f/f^ mice) with transgenic mice expressing Cre recombinase under the control of the CAG promoter (CAGCre mice). Liver-specific Mtu1 KO mice were generated by crossing Mtu1^f/f^ mice with transgenic mice carrying Cre recombinase under the control of the albumin promoter (AlbCre mice). Mtu1^f/f^ mice, CAGCre transgenic mice and AlbCre transgenic mice were backcrossed with C57BL6/J mice for at least seven generations to control the genetic background. Mice were housed at 25°C with 12 h light and 12 h dark cycles. Unless otherwise indicated, we sacrificed 3- to 5-week-old male mice for all experiments in Figs 1–7. All animal procedures were approved by the Animal Ethics Committee of Kumamoto University (Approval ID: A27-037). Detailed information on genotyping can be found in the “Supplemental Methods”.

### Primary hepatocyte culture

Primary hepatocytes were isolated from Mtu1^f/f^ and Mtu1^LKO^ mice by perfusion of collagenase (Worthington Biochemical Corporation, Lakewood, NJ) following the manufacturer’s instructions. Isolated hepatocytes were cultured in high glucose DMEM (Thermo Fisher Scientific, Waltham, MA) supplemented with 10% fetal bovine serum (Hyclone, GE Healthcare, NJ) for 3 h. Subsequently, the culture medium was replaced with DMEM without serum for 14 h. All experiments using primary hepatocytes were performed within 24 h after isolation.

### Gene expression analysis

Total RNA was isolated from fresh liver samples using TRIzol reagent (ThermoFisher Scientific, USA) following the manufacturer’s instructions. cDNA was synthesized from 100 ng of total RNA using the PrimerScript RT-PCR kit (TAKARA, Tokyo, Japan) and subjected to quantitative PCR (SYBR Premix Ex Taq II, TAKARA) using a 7300 Real Time PCR System (Thermo Fisher Scientific, CA). The sequence information is included in the Supplemental Methods.

### Western blotting

The mitochondrial fraction was isolated from the livers of conditional Mtu1 knockout mice or HeLa cells using MOPS buffer as described previously. Ten micrograms of each sample were loaded into 12% SDS-PAGE gels and transferred to PVDF membranes. Mitochondrial and cellular proteins were detected using the proper antibodies as described in the supporting information.

### Statistics

The data were analyzed using GraphPad Prism 6 software. An unpaired Student’s *t*-test was used to test the differences between two groups. A 2-tailed *P*-value of 0.05 was considered significant. The results are shown as the means ± S.E.M.

Detail methods are provided in the “Supporting Information”.

## Supporting Information

S1 Fig Morphological examination of liver-specific Mtu1 knockout mice. (A) Strategy for the generation of liver-specific Mtu1 knockout mice (Mtu1^LKO^: LKO). Mice carrying the floxed *Mtu1* gene were used as a control (Mtu1^Flox^: Flox). (B) Representative male Mtu1^LKO^ and Mtu1^Flox^ mice at 6 weeks of age. (C) Representative genotyping results of the Mtu1^LKO^ and Mtu1^Flox^ mice shown in (B). (D) Representative H&E staining of liver sections of control mice (Flox) and Mtu1^LKO^ mice (LKO). Bars = 0.2 mm.
(TIF)

S2 Fig Relative amount of modifications at 34U. (A-C) Individual mt-tRNA^Lys^, mt-tRNA^Glu^ and mt-tRNA^Gln^ were isolated from Mtu1^Flox^ and Mtu1^LKO^ mice and subjected to mass spectrometry analysis. Representative mass chromatograms of mt-tRNA fragments containing τm^5^U, τm^5^s^2^U, s^2^U or U at position 34 were shown. Arrowheads indicate traces of fragments containing s^2^ modification in LKO mice. (D) The relative amounts of τm^5^U, τm^5^s^2^U, s^2^U and U at position 34 were calculated from the peak areas and plotted.
(TIF)

S3 Fig Sulfur-containing tRNA modifications in Mtu1^LKO^ mice. Total RNA was isolated from the livers of 3-week-old Mtu1^LKO^ and Mtu1^Flox^ mice. RNA was digested and subjected to mass spectrometry. The levels of mcm^5^s^2^U and ms^2^i^6^A modifications were normalized to the levels of mcm^5^U and i^6^A modifications, respectively. n = 4 each.
(TIF)

S4 Fig Glutathione and glutathione disulfide levels in Mtu1^LKO^ mice. (A) Relative levels of glutathione disulfide (GSSG) in liver tissues of 3-week-old Mtu1^LKO^ and Mtu1^flox^ mice (LKO: 61% versus Flox mice). n = 4; *P* = 0.059. (B) Relative levels of glutathione (GSH) (LKO: 117% versus Flox mice). n = 4; *P* = 0.4. (C) Relative GSSG/GSH ratios (LKO: 50% versus Flox mice). n = 4; *P* = 0.057. (D) Relative levels of cysteine (LKO: 92% versus Flox mice). n = 4; *P* = 0.71.
(TIF)

S5 Fig Codon usage in mouse and human mitochondrial genes. (A) Codon numbers of Lys (AAA, AAG), Glu (GAA, GAG) and Gln (CAA, CAG) in mouse and human mitochondrial mRNAs. Yellow columns represent the 4 transcripts that exhibited normal translation in Mtu1-deficient hepatocytes. (B) Codon frequencies of AAA/GAA/CAA and AAG/GAG/CAG in mouse and human mitochondrial mRNAs. The 4 transcripts shown in yellow letters correspond to the 4 transcripts that exhibited normal translation in Mtu1-deficient hepatocytes. (C) Correlation of the number of AAA/GAA/CAA codons with the total length of the transcripts. *P* = 0.001.
(TIF)

S1 Text Supplemental Methods. (DOCX)
