# Supplementary figures and images for: Mtu1-Mediated Thiouridine Formation of Mitochondrial tRNAs Is Required for Mitochondrial Translation and Is Involved in Reversible Infantile Liver Injury
Source: PLoS Genet. 2016 Sep 30;12(9):e1006355. doi: 10.1371/journal.pgen.1006355 (PMC5045200; doi:10.1371/journal.pgen.1006355)

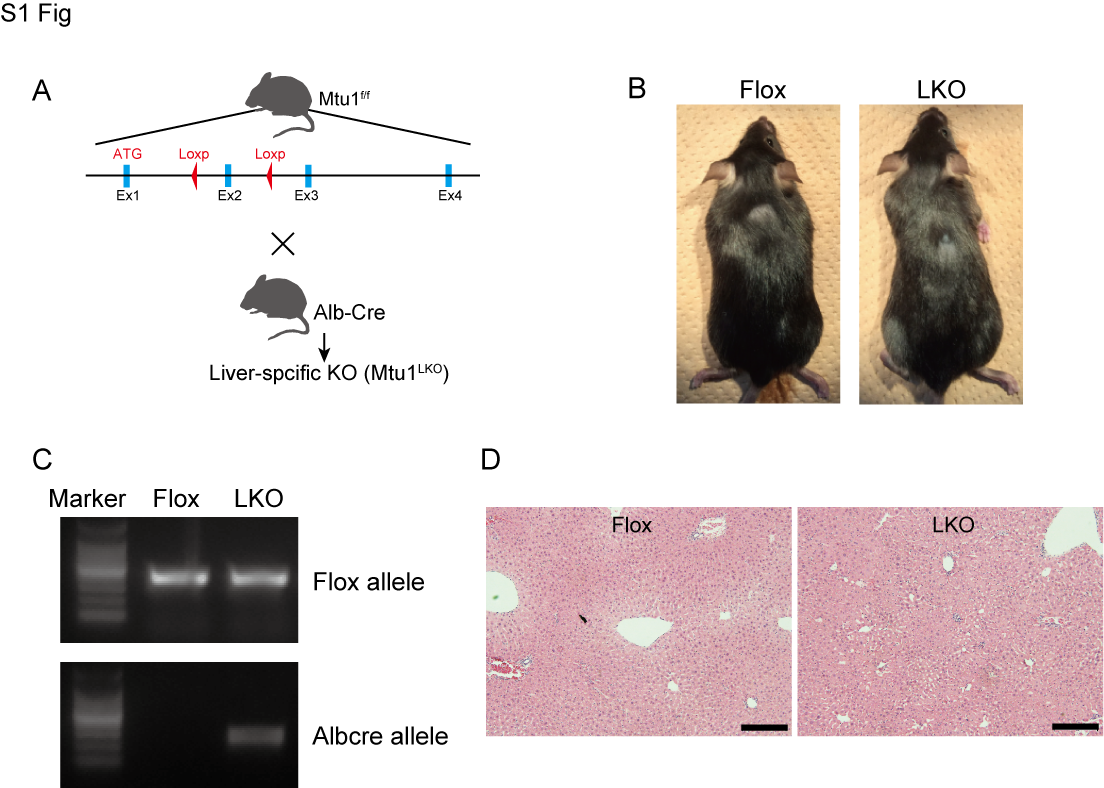

Supplement: S1 Fig — (A) Strategy for the generation of liver-specific Mtu1 knockout mice (Mtu1LKO: LKO). Mice carrying the floxed Mtu1 gene were used as a control (Mtu1Flox: Flox). (B) Representative male Mtu1LKO and Mtu1Flox mice at 6 weeks of age. (C) Representative genotyping results of the Mtu1LKO and Mtu1Flox mice shown in (B). (D) Representative H&E staining of liver sections of control mice (Flox) and Mtu1LKO mice (LKO). Bars = 0.2 mm. (TIF) [file pgen.1006355.s001.tif]

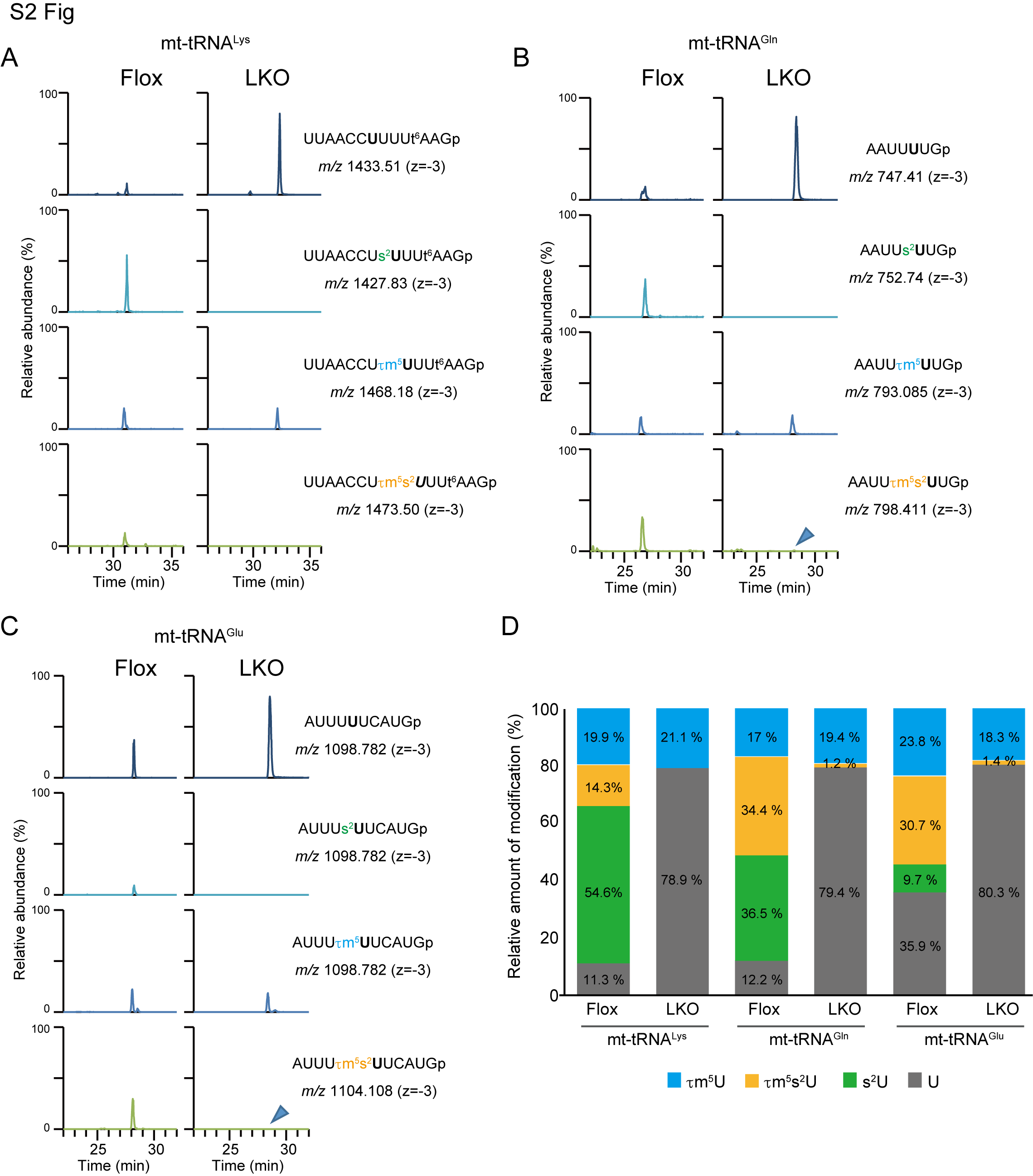

Supplement: S2 Fig — (A-C) Individual mt-tRNALys, mt-tRNAGlu and mt-tRNAGln were isolated from Mtu1Flox and Mtu1LKO mice and subjected to mass spectrometry analysis. Representative mass chromatograms of mt-tRNA fragments containing τm5U, τm5s2U, s2U or U at position 34 were shown. Arrowheads indicate traces of fragments containing s2 modification in LKO mice. (D) The relative amounts of τm5U, τm5s2U, s2U and U at position 34 were calculated from the peak areas and plotted. (TIF) [file pgen.1006355.s002.tif]

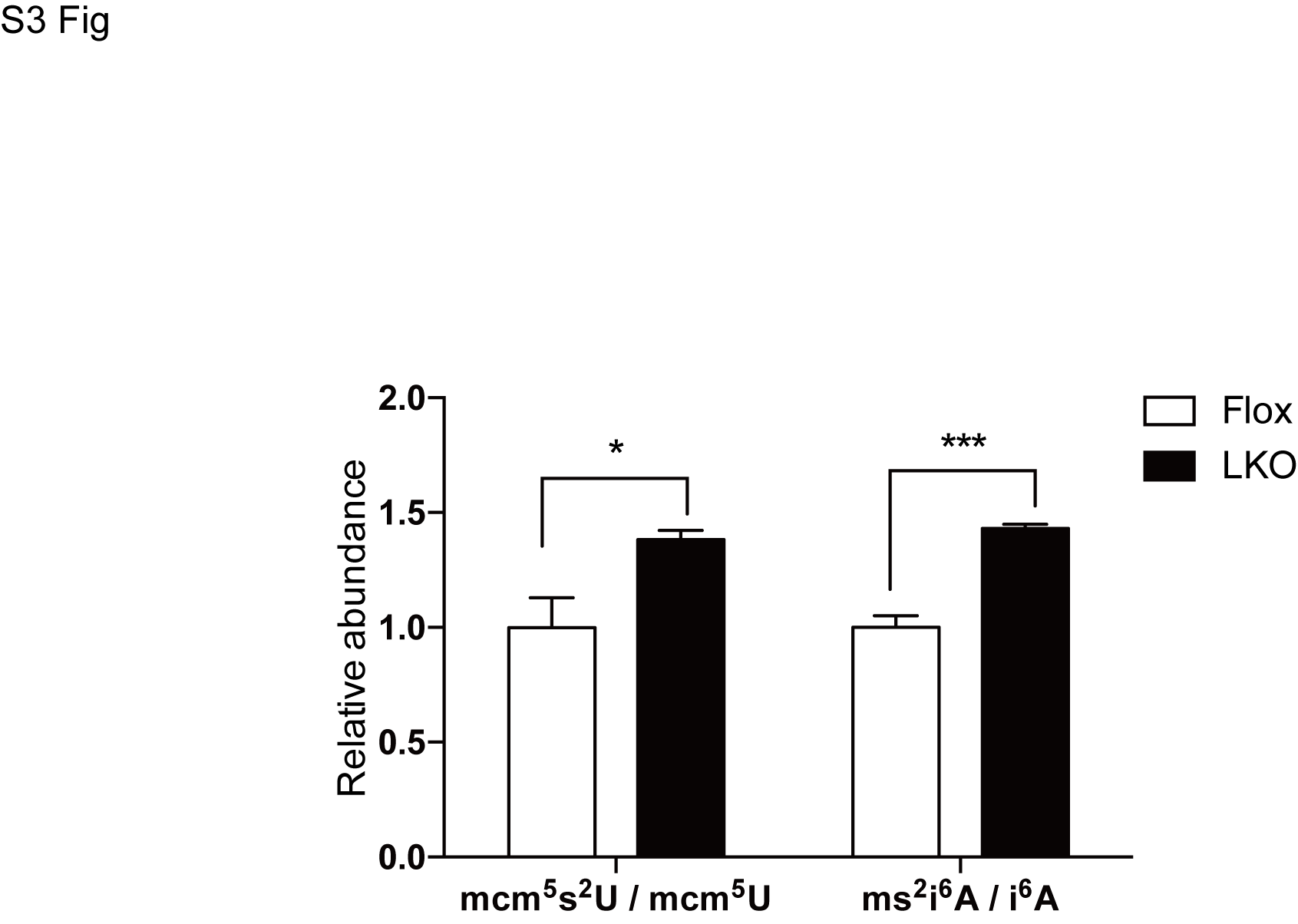

Supplement: S3 Fig — Total RNA was isolated from the livers of 3-week-old Mtu1LKO and Mtu1Flox mice. RNA was digested and subjected to mass spectrometry. The levels of mcm5s2U and ms2i6A modifications were normalized to the levels of mcm5U and i6A modifications, respectively. n = 4 each. (TIF) [file pgen.1006355.s003.tif]

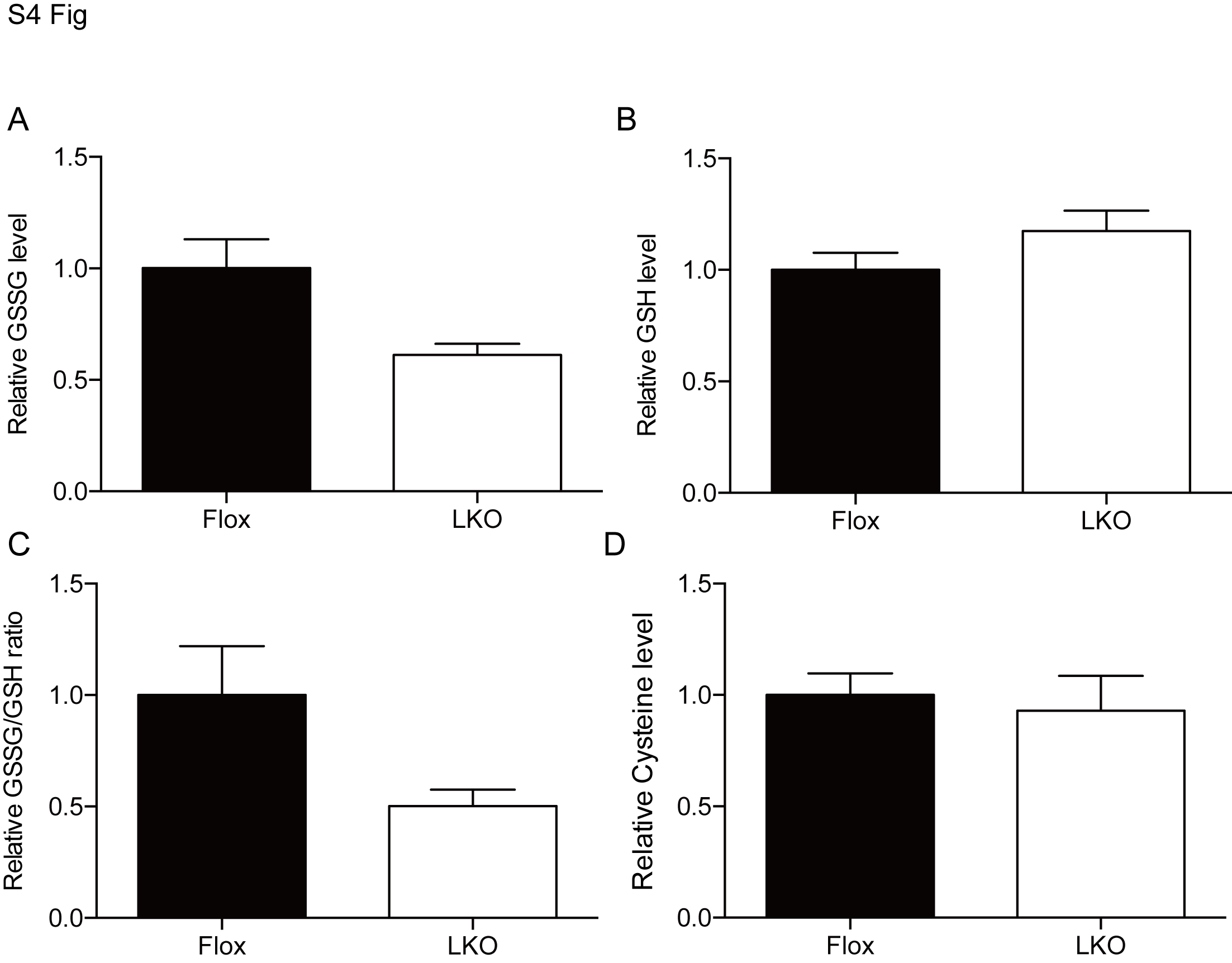

Supplement: S4 Fig — (A) Relative levels of glutathione disulfide (GSSG) in liver tissues of 3-week-old Mtu1LKO and Mtu1flox mice (LKO: 61% versus Flox mice). n = 4; P = 0.059. (B) Relative levels of glutathione (GSH) (LKO: 117% versus Flox mice). n = 4; P = 0.4. (C) Relative GSSG/GSH ratios (LKO: 50% versus Flox mice). n = 4; P = 0.057. (D) Relative levels of cysteine (LKO: 92% versus Flox mice). n = 4; P = 0.71. (TIF) [file pgen.1006355.s004.tif]

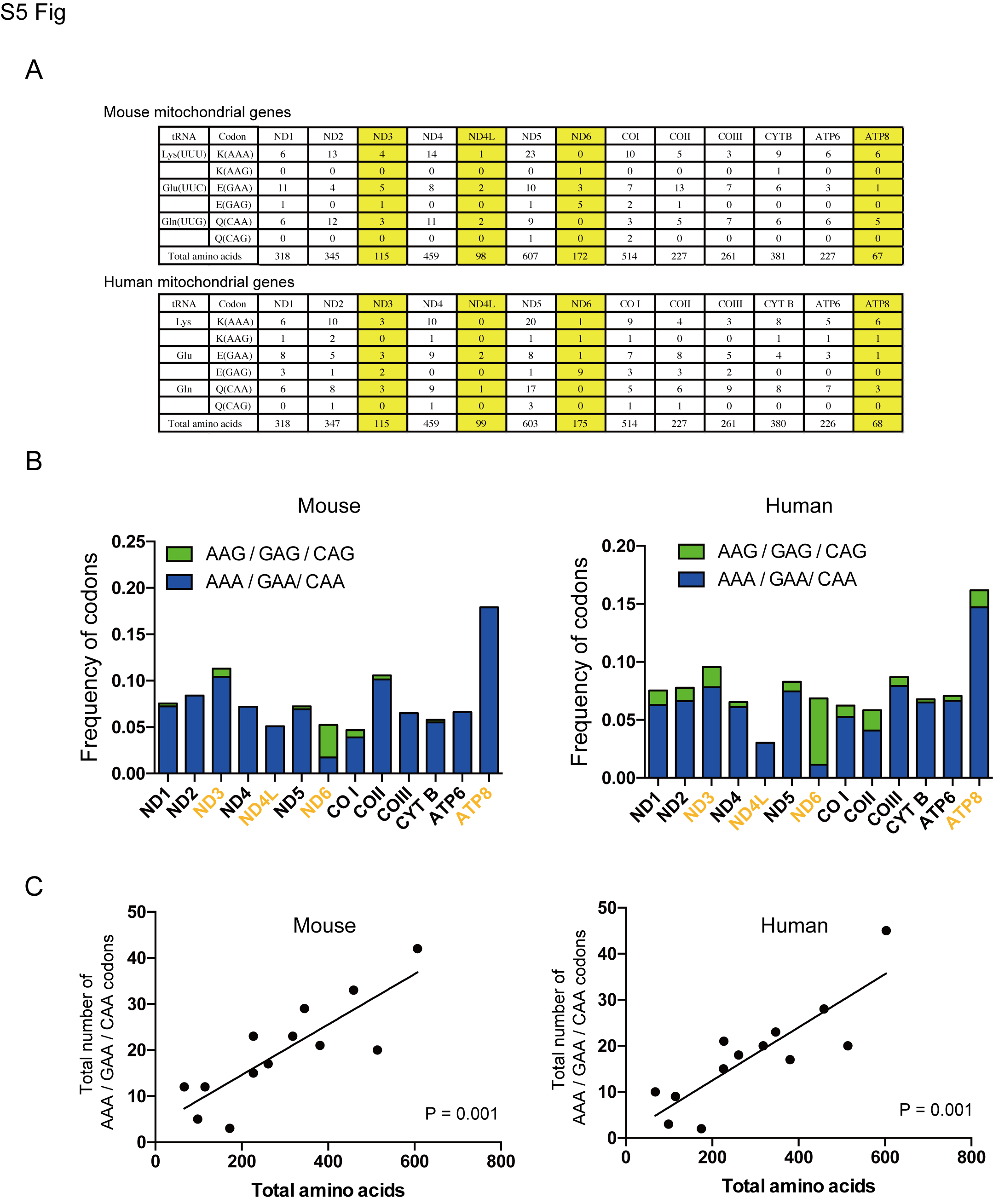

Supplement: S5 Fig — (A) Codon numbers of Lys (AAA, AAG), Glu (GAA, GAG) and Gln (CAA, CAG) in mouse and human mitochondrial mRNAs. Yellow columns represent the 4 transcripts that exhibited normal translation in Mtu1-deficient hepatocytes. (B) Codon frequencies of AAA/GAA/CAA and AAG/GAG/CAG in mouse and human mitochondrial mRNAs. The 4 transcripts shown in yellow letters correspond to the 4 transcripts that exhibited normal translation in Mtu1-deficient hepatocytes. (C) Correlation of the number of AAA/GAA/CAA codons with the total length of the transcripts. P = 0.001. (TIF) [file pgen.1006355.s005.tif]
