# Supplementary material for: Mtu1-Mediated Thiouridine Formation of Mitochondrial tRNAs Is Required for Mitochondrial Translation and Is Involved in Reversible Infantile Liver Injury
Source: PLoS Genet. 2016 Sep 30;12(9):e1006355. doi: 10.1371/journal.pgen.1006355 (PMC5045200; doi:10.1371/journal.pgen.1006355)
Supplement: S1 Text — (DOCX) [file pgen.1006355.s006.docx]

**Supplemental Methods**

**Genotyping**

To genotype the Mtu1^f/f^ and Mtu1^LKO^ mice, genomic DNA was extracted from small pieces of tails clipped from 3-week-old mice using phenol/chloroform precipitation as described elsewhere. Ten nanograms of DNA was subjected to PCR for genotyping. The primers to detect the Flox allele and Albcre allele were as follows:

Flox/WT allele forward: 5’-TTATAGGAACTGGCTGTCTAGTTGG

Flox/WT allele reverse: 5’-CAAACTTTACCTTCCTGAGATGGAT

Cre allele forward: 5’-ACATGTTCAGGGATCGCCAG

Cre allele reverse: 5’-TAACCAGTGAAACAGCATTGC

For genotyping Mtu1^+/+^, Mtu1^+/-^ and Mtu1^-/-^ embryos at E9, a pair of male and female Mtu1^+/-^ mice were mated for 3 h (5 pm ~ 8 pm). The following morning was counted as developmental stage E0.5. The embryos at stage E9 were removed, and the yolk sacs were used for extraction of embryonic DNA. Briefly, the yolk sacs were lysed in 180 μl of 50 mM NaOH at 95 ˚C for 10 min. The extracts were neutralized by the addition of 20 μl of 1 M Tris-HCl, pH 8.0. After a brief centrifugation at 12,000 rpm for 10 min, 1 μl of the extract was subjected to PCR using KOD FX DNA polymerase (TOYOBO Life Science, Tokyo, Japan) following the manufacturer’s instructions. The primers to detect the wild-type allele and knockout allele were as follows:

Flox/WT allele forward: 5’-TTATAGGAACTGGCTGTCTAGTTGG

Flox/WT allele reverse: 5’-CAAACTTTACCTTCCTGAGATGGAT

Knockout allele forward: 5’-TTAAATGTTTGGTGTTGAGAATTGA

Knockout allele reverse: 5’- CAAACTTTACCTTCCTGAGATGGAT

**Histological examination**

Liver tissues and embryos were fixed overnight using 4% paraformaldehyde (Wako, Tokyo, Japan). The liver tissues were embedded in paraffin and sectioned to a 3-μm thickness. The sections were stained with Mayer’s hematoxylin and eosin (H&E). Masson’s trichrome staining was performed to examine hepatic fibrosis as described elsewhere. To examine the blood vessel network, the embryos were permeabilized with 0.1% Triton X-100 in phosphate buffer and stained with an anti-PECAM1 antibody (Clone MEC 13.3, BD Bioscience, Franklin Lakes, NJ) diluted 1:500 in 2% skim milk (Wako, Tokyo, Japan). For examination with an electron microscope, liver tissues were fixed in a solution containing 2% paraformaldehyde and 2% glutaraldehyde. Random sections were obtained from two livers per group. The mitochondrial areas in images taken at 5000x magnification were calculated using ImageJ software.

**Mitochondrial translation**

Labeling of mitochondrial protein synthesis was evaluated as described previously [1]. Primary hepatocytes were isolated and plated in 10-cm dishes at 80~90% confluency. Fourteen hours after plating, hepatocytes were washed with DMEM without methionine and cysteine (Thermo Fisher Scientifc, Waltham, MA). To inhibit cytosolic translation, emetine (Sigma, St. Louis, MO) was added to the medium at 100 μg/mL for 6 min. Subsequently, 100 μCi of ^35^S-methionine/cysteine (Tran^35^S-LABEL, MP Biomedicals, Santa Ana, CA) was added to the hepatocytes for 1 h. Metabolic labeling was terminated by adding cold phosphate buffer containing excess methionine and cysteine. The mitochondria were then isolated from the primary hepatocytes as described previously. Ten micrograms of mitochondrial protein were subjected to SDS-PAGE using a 15~20% gradient gel. The gel was dried and exposed to Imaging Plate (Fujifilm, Tokyo, Japan).

**Blue-Native PAGE and western blotting**

Mitochondria were isolated from the liver tissues of Mtu1^f/f^ and Mtu1^LKO^ mice using MOPS buffer (250 mM sucrose, 20 mM MOPS, 1 mM EDTA, pH 7.2, protease inhibitor cocktail (Roche, Basel, Switzerland). The livers were minced to small pieces and then homogenized in a Teflon homogenizer for 20 strokes at 1,200 rpm on ice. The homogenate was centrifuged at 800 g for 15 min at 4 ˚C, and the supernatant was further centrifuged at 8,000 g for 15 min. The mitochondrial sediment was resuspended in MOPS buffer and adjusted to 1 mg/mL using a BCA protein assay kit (Thermo Fisher Scientifc, Waltham, MA). Subsequently, 125 μg of fresh mitochondria was resuspended in 40 μl of solubilizing buffer (50 mM Bis-Tris, 1 M aminocaproic acid). The mitochondria were solubilized in 6 μl of 10% n-dodecyl β-D-maltoside (Sigma). Each mitochondria sample was centrifuged at 100,000 g for 15 min at 4 ℃. The supernatant was mixed with 3 μl of 5% Serva G (Sigma), and 10 μl of the sample was subjected to blue-native PAGE using a 3–12% Bis-Tris Novex NativePAGE gel (Thermo Fisher Scientifc). The protein level of the assembled respiratory Complexes I-IV was assessed by western blotting with the following commercial antibodies: anti-NDUFB8 (ab110242, Abcam), anti-SDHA (ab137040, Abcam), anti-UQCRC2 (ab14745, Abcam), anti-MTCO1 (ab14705, Abcam), and anti-ATP5A (ab14748, Abcam). All primary antibodies were diluted 1:20,000 in 5% skim milk.

For standard western blotting, 10 μg of mitochondrial proteins was loaded on a 12% SDS-PAGE gel. The following commercial antibodies were used in this experiment: anti-Prohibitin (ab75771, Abcam), anti-AFG3L2 (ab68023, Abcam), anti-LONP1 (ab103809, Abcam), anti-Mitofusin 1 (MFN1) (ab57602, Abcam), anti-PARKIN (ab15954, Abcam), anti-phospho-mTOR (#5536, Cell Signaling, Danvers, MA), anti-mTOR (#2983, Cell Signaling), anti-phospho-p44/42 MAPK (#4370, Cell Signaling), anti-p44/42 (#4695, Cell Signaling) and anti-β−Actin (Medical & Biological Laboratories, Nagoya, Japan). All the primary antibodies were diluted 1:1,000. PVDF membrane was stained using PageBlue Protein Staining Solution (Thermo Fisher Scientific) according to the manufacturer’s instruction.

An OxyBlot protein oxidation detection kit (Millipore, Billerica, MA) was used to detect protein carbonylation in the mitochondria of liver tissue following the manufacturer’s protocol. Briefly, 5 μl of 4 mg/ml mitochondrial proteins was mixed with 5 μl of 12% SDS, followed by the addition of either 10 μl of DNPH solution or 10 μl of derivatization solution for the negative control. Ten microliters of the final sample were directly subjected to SDS-PAGE followed by western blotting using the anti-DNP antibody provided in the kit.

**Complex activities**

The enzymatic activity of mitochondrial respiratory complexes (Complexes I~IV and citrate synthase) was determined using mitochondria obtained from Mtu1^f/f^ or Mtu1^LKO^ mice as described previously. Ten to twenty microliters of liver mitochondria were used in the enzymatic assays as described previously [1-2].

**Analysis of tRNA modification by mass spectrometry**

To examine mt-tRNA modifications, total RNA was isolated from the livers of Mtu1^f/f^ or Mtu1^LKO^ mice using TRIzol (Thermo Fisher Scientific). Individual mt-tRNA^Gln^, mt-tRNA^Glu^ or mt-tRNA^Lys^ was isolated using the RCC method and subjected to mass spectrometry analysis as described previously [3-5]. The sequences of 5'-amino-modified oligo DNAs were complementary to the sequence of mt-tRNA^Gln^, mt-tRNA^Glu^ or mt-tRNA^Lys^ (NC_005089). The sequences were as follows:

mt-tRNA^Gln^: 5’-TCAAAATTCTCCGTGCTACCTAAACACCTTATCCT

mt-tRNA^Glu^: 5’- ACAGCATTCAACTGCGACCAATGACATGAAAAATCATCGT

mt-tRNA^Lys^: 5’-AGGTCTCTAACTTTAACTTAAAAGGTTAACGCTCT

**Analysis of glutathione and glutathione disulfide**

Hepatic glutathione (GSH) and glutathione disulfide (GSSG) levels were examined using mass spectrometry (Agilent, 6460) as described previously [6]. Fresh liver tissues were snap-frozen on dry ice and stored at -80 ˚C until processing for analysis. Before the metabolites were extracted from the liver samples, monobromobimane (mBBr; Thermo Fisher Scientific) was dissolved in methanol at a final concentration of 5 mM. The tissue sample was weighed, and mBBr/methanol was added at a concentration of 10 mg/ml. The tissue was quickly homogenized using a TissueRuptor (Qiagen, Hilden, Germany) and incubated at 37 ˚C for 15 min. The samples were centrifuged at 10,000 x g for 10 min to precipitate insoluble materials. The supernatant was mixed with water to result in a 10-fold dilution, and the sample was directly subjected to mass spectrometry analysis using the multiple reaction monitoring (MRM) method. The parameters for the MRM method were described previously [6].

**Blood biochemistry**

Plasma AST and ALT levels were measured using an Aspartate Assay kit (Abcam) following the manufacturer’s instructions. The plasma lactate level was measured using a Lactate Colorimetric Assay kit (BioVision, Milpitas, CA) following the manufacturer’s instructions. Serum biochemistry analysis was performed using Clinical Biochemistry Analyzer (JCA-BM6050, JEOL, Tokyo, Japan).

**mtDNA Quantification**

The relative mtDNA content was examined using quantitative PCR as described previously [1]. Briefly, DNA was extracted from the tissues of WT and KO mice using a QIAamp DNA Mini Kit (Qiagen). The DNA was directly subjected to qPCR to amplify the CytB gene (forward: 5’-GCTTTCCACTTCATCTTACCATTT, reverse: 5’-TGTTGGGTTGTTTGATCCTG) for mtDNA and the beta-Actin gene (forward: 5’-GGAAAAGAGCCTCAGGGCAT, reverse: 5’-GAAGAGCTATGAGCTGCCTGA for nuclear DNA, respectively. The qPCR was performed using a SYBR Premix Ex Taq Kit (Takara, Japan) and the ABI PRISM 7300 Real-Time PCR System (Life Technologies) according to the manufacturer’s protocol.

**Primer list for quantitative PCR**

For the analysis of gene expression by heat map (related to Fig 2F and Fig 7E), a Z transformation was applied to the results to calculate Z scores and to construct a heat map [7].

|  | Forward Primer | Reverse Primer |
| --- | --- | --- |
| Oxidative response genes |  |  |
| Alb | CAAGAGTGAGATCGCCCATCG | TTACTTCCTGCACTAATTTGGCA |
| Alox12 | GTTCCACACATCCGTTACACT | CCGAGTAAGCAACTGAACATGG |
| Angptl7 | TGACTGTTCTTCCCTGTACCA | CAAGGCCACTCTTACGTCTCT |
| Aox1 | GAGGAAGAATCTCCGACTCACA | TGGTGACTGCTGTACCATGTAG |
| ApoE | CTGACAGGATGCCTAGCCG | CGCAGGTAATCCCAGAAGC |
| Atox1 | ATGCCGAAGCACGAGTTCTC | ATGCAGACCTTCTTGTTGGGC |
| Bnip3 | CTGGACGAAGTAGCTCCAAGA | ACAGAGTGCTGTTTTTCTCGC |
| Ccl5 | GCTGCTTTGCCTACCTCTCC | TCGAGTGACAAACACGACTGC |
| Ccs | TGTGCGTTGGAGTTTGCAGT | GCCTCCCTGTGCTTTCCAG |
| Csde1 | GATGTGGAAGGGAATGTTCAGC | ATTACGAGCACTTACAGCACC |
| Cyba | TCACCAGGAATTACTACGTCCG | GCTGCCAGCAGATAGATCACA |
| Dhcr24 | CTCTGGGTGCGAGTGAAGG | TCCCGGACCTGTTTCTGGAT |
| Dusp1 | AGTACCCCTCTCTACGATCAGG | CGAGAAGCGTGATAGGCACTG |
| Ephx2 | GCGTTCGACCTTGACGGAG | TGTAGCTTTCATCCATGAGTGGT |
| Foxm1 | ATCACGGAGACGTTGGGAC | CCACTGGATATTGGTTAAGCTGT |
| Glrx2 | ATCGTCGTTTTGGGGGAAGTC | GGAACAGTAAGAGCAGGATGTTT |
| Gpx1 | GTGCAATCAGTTCGGACACCA | CACCAGGTCGGACGTACTTG |
| Gpx2 | GAGCTGCAATGTCGCTTTCC | TGGGTAAGACTAAAGGTGGGC |
| Gpx3 | CCTTTTAAGCAGTATGCAGGCA | GGGGAGTATCTCCGAGTTCTC |
| Gpx4 | GATGGAGCCCATTCCTGAACC | CCCTGTACTTATCCAGGCAGA |
| Gpx6 | GCCCAGAAGTTGTGGGGTTC | TCCATACTCATAGACGGTGCC |
| Gpx7 | TCCGAGCAGGACTTCTACGAC | TCTCCCTGTTGGTGTCTGGTT |
| Gsr | GACACCTCTTCCTTCGACTACC | CACATCCAACATTCACGCAAG |
| Gss | CAAAGCAGGCCATAGACAGGG | AAAAGCGTGAATGGGGCATAC |
| Gstz1 | TTACTTCCGGCTTTAACGCTC | TGGGGTACTAAGCACACATCA |
| Gtf2i | GCCTGCCGAAGATGAAGAG | AACAAACACGTCGGTCTCATAC |
| Mbl2 | TGACAGTGGTTTATGCAGAGAC | CGTCACGTCCATCTTTGCC |
| Mgst3 | TTATTGGGCGAGTCCTTTACGC | CGTAGCCTAAGCCTGGTCTG |
| Mpv17 | GGAGGCTGGTACAAAGTTTT | CATTCCATTGAGTATCCCGACC |
| Msra | CACGCAATCCCACCTACAAAG | CGGGTCGTGATTCTCCCAG |
| Mt3 | ACCTGCCCCTGTCCTACTG | CCTTGGCACACTTCTCACATC |
| Ncf1 | ACACCTTCATTCGCCATATTGC | TCGGTGAATTTTCTGTAGACCAC |
| Ncf2 | GCTGCGTGAACACTATCCTGG | AGGTCGTACTTCTCCATTCTGTA |
| Nme5 | AAAACCCTAGCCCTTATCAAGC | AGGTGTAGTTTCCGTCTCTGAA |
| Nudt1 | TTCTCCTGGGCATGAAGAAGAG | AGGAGAGCCCACAAATTCAAAC |
| Oxr1 | AAGCGACCCCAGTGAACTCT | TGGATAGCCAATGGTTCTTGGT |
| Oxsr1 | CATTGTGGCAAAGGGGGAAC | TGACGCCGAAATCTGCAATCT |
| Pdlim1 | TCGATGGGGAAGATACCAGCA | TCTGTTCAGACCTGGATACTGTG |
| Ipcef1 | AAGCAGATGGATTTGTCAACCTT | TCATCCTTTGTGATGGATTCCTG |
| Pnkp | CGAAACCAAGTGGAGCTGATT | CAGTCCTGGCTTCAACTCCTG |
| Prdx1 | AATGCAAAAATTGGGTATCCTGC | CGTGGGACACACAAAAGTAAAGT |
| Prdx2 | CACCTGGCGTGGATCAATACC | GACCCCTGTAAGCAATGCCC |
| Prdx3 | TGCTCGTCATGCAAGTGCTATT | CAGGGGTGTGGAAAGAGGAACT |
| Prdx4 | CTCAAACTGACTGACTATCGTGG | CGATCCCCAAAAGCGATGATTTC |
| Prdx5 | GGCTGTTCTAAGACCCACCTG | GGAGCCGAACCTTGCCTTC |
| Prdx6 | CGCCAGAGTTTGCCAAGAG | TCCGTGGGTGTTTCACCATTG |
| Prex1 | TTTAACCAGGTCGATTCCATCCA | CGGACCGTGCATTCCTCTTT |
| Prnp | ATGGCGAACCTTGGCTACTG | CCTGAGGTGGGTAACGGTTG |
| Ptgs1 | ATGAGTCGAAGGAGTCTCTCG | GCACGGATAGTAACAACAGGGA |
| Ptgs2 | TGAGCAACTATTCCAAACCAGC | GCACGTAGTCTTCGATCACTATC |
| Pxdn | TAGAAACGCTGGACCCTGAAT | CTAAGTGCGTGATACGGTTGT |
| Rnf7 | ATGTGGAGCTGGGACGTTG | GAGGGCAGCGATTGTTCTGT |
| Scara3 | TGACAGGGATGTACTGTGTGT | TGCAAAGATAGGTTCTTCTGGC |
| Vimp | GACCGAGAGCCTGCGATTC | AGCCCTCAGTCGAAGGGAG |
| Sepp1 | AGCTCTGCTTGTTACAAAGCC | CAGGTCTTCCAATCTGGATGC |
| Sftpd | ACGTGGACTAAGTGGACCTCC | CCTTTTGCCCCTGTAGATCCTT |
| Sgk2 | TCCAGCCCAGTTGGAGTTC | CCGTAGTTCCCTTTGCCAATG |
| Srxn1 | CACGGTGCACAACGTACCAATC | GGCAGCCCCCAAAGGAATAG |
| Stk25 | ATATCACCCGCTACTTCGGCT | GGTGGCAATATAGGTCTCTTCCA |
| Ttn | TGTCGTTGCTGCTGTTGATATG | CTTGCGTTGTCACCATCACTTC |
| Txnrd1 | GGGTCCTATGACTTCGACCTG | AGTCGGTGTGACAAAATCCAAG |
| Txnrd2 | GATCCGGTGGCCTAGCTTG | TCGGGGAGAAGGTTCCACAT |
| Metabolic genes |  |  |
| Gck | TGAGCCGGATGCAGAAGGA | GCAACATCTTTACACTGGCCT |
| Pfk | GGAGGCGAGAACATCAAGCC | CGGCCTTCCCTCGTAGTGA |
| G6pc | CGACTCGCTATCTCCAAGTGA | GTTGAACCAGTCTCCGACCA |
| Fbp1 | CACCGCGATCAAAGCCATCT | AGGTAGCGTAGGACGACTTCA |
| Pcx | CTGAAGTTCCAAACAGTTCGAGG | CGCACGAAACACTCGGATG |
| Gys | ACCAAGGCCAAAACGACAG | GGGCTCACATTGTTCTACTTGA |
| Pyg | GAGAAGCGACGGCAGATCAG | CTTGACCAGAGTGAAGTGCAG |
| Hmgcs1 | AACTGGTGCAGAAATCTCTAGC | GGTTGAATAGCTCAGAACTAGCC |
| Hmgcs2 | GAAGAGAGCGATGCAGGAAAC | GTCCACATATTGGGCTGGAAA |
| Hmgcl | CCGGCATCAACTACCCAGTC | GCGCTGGAAACTCTCCTCTAT |
| Bdh1 | ACAAGACACACGCTGTTGTTT | CTCTTCAAGCTGTCCAGTTCC |
| Acly | CATCGGCGTTGCGTTTGTGG | GCCCATACTCCTTCCTAGCA |
| Eovl6 | GCTCAGCAAAGCACCCGAACTAGG | GGAGTACCAGGAGTACAGGAGCACAGTG |
| Scd1 | CAGCCTGTTCGTTAGCACCTTCTTG | CACTGGCAGAGTAGTCGAAGGGGAAG |
| Gpam | CTTCAGAGGCTTCTAGGTCCCCTG | CGTTCTTCACGAGACAGTATGTGGC |
| Srebf1 | CTGCCCGGACACACCAGCTC | TGCCCAGGAGCCGACAGGAA |
| Ppara | GGCGTTTCCTGAGACCCTCG | GGCTCTCTGTGTCCACCATG |
| Cpt1a | CTCCGCCTGAGCCATGAAG | CACCAGTGATGATGCCATTCT |
| Cpt2 | CAGCACAGCATCGTACCCA | TCCCAATGCCGTTCTCAAAAT |
| Acc1 | ATGGGCGGAATGGTCTCTTTC | TGGGGACCTTGTCTTCATCAT |
| Acc2 | CGCTCACCAACAGTAAGGTGG | GCTTGGCAGGGAGTTCCTC |
| Pgc1a | TATGGAGTGACATAGAGTGTGCT | CCACTTCAATCCACCCAGAAAG |
| Fgf21 | CTGCTGGGGGTCTACCAAG | CTGCGCCTACCACTGTTCC |
| Reference gene |  |  |
| 18S | GTAACCCGTTGAACCCCATT | CCATCCAATCGGTAGTAGCG |
| Mitochondrial DNA-encoded genes |  |  |
| CoI | ATGAGCAAAAGCCCACTTCG | AAGGCCCAGGAAATGTTGAG |
| CoII | TTCATCTGAAGACGTCCTCCAC | TGATTTAGTCGGCCTGGGATG |
| CoIII | TCATGGCTACTGGATTCCATGG | TCATGCTGCGGCTTCAAATC |
| Atp6 | AAAGCTCACTTGCCCACTTC | GGACTGCTAATGCCATTGGTTG |
| Atp8 | GGCACCTTCACCAAAATCAC | TTGTTGGGGTAATGAATGAGG |
| Cytb | TTGCATACGCCATTCTACGC | TAGGCTTCGTTGCTTTGAGG |
| Nd1 | TCCCCATTCTAATCGCCATAGC | ATGGCGTCTGCAAATGGTTG |
| Nd2 | ATGAGTAGGCCTGGAATTCAGC | TTGAGGCTGTTGCTTGTGTG |
| Nd3 | ATTCGACCCTACAAGCTCTGC | CATGGTAGTGGAAGTAGAAGAGC |
| Nd4 | CAACACACACCTTAGACGCTTC | GCTTCAACATGGGCTTTTGG |
| Nd4l | AAGCTCCATACCAATCCCCATC | TGGACGTAATCTGTTCCGTACG |
| Nd5 | ACCCACGCATTCTTCAAAGC | AGGCTTCCGATTACTAGGCATG |
| Nd6 | ATGTTGGAAGGAGGGATTGGG | ACCCGCAAACAAAGATCACC |
| Rnr1 | TCGGCGTAAAACGTGTCAAC | AGTTTGGGTCTTAGCTGTCGTG |
| Rnr2 | AAACCCCGAAACCAAACGAG | AGCTCGTTAGGCTTTTCACC |

**Supplemental references**

1. Wei FY, Zhou B, Suzuki T, Miyata K, Ujihara Y, Horiguchi H, et al. Cdk5rap1-mediated 2-methylthio modification of mitochondrial tRNAs governs protein translation and contributes to myopathy in mice and humans. Cell Metab 2015;21:428-442.

2. Spinazzi M, Casarin A, Pertegato V, Salviati L, Angelini C. Assessment of mitochondrial respiratory chain enzymatic activities on tissues and cultured cells. Nat Protoc. 2012;7:1235-1246.

3. Miyauchi K, Ohara T, and Suzuki T. Automated parallel isolation of multiple species of non-coding RNAs by the reciprocal circulating chromatography method. Nucleic Acids Res*.* 2007;35:e24.

4. Miyauchi K, Kimura S, and Suzuki T. A cyclic form of *N*^6^-threonylcarbamoyladenosine as a widely distributed tRNA hypermodification. Nat Chem Biol*.* 2013;9:105-111.

5. Suzuki T, Suzuki T. (2014). A complete landscape of post-transcriptional modifications in mammalian mitochondrial tRNAs. Nucleic Acids Res. 2014;42:7346-7357.

6. Ida T, Sawa T, Ihara H, Tsuchiya Y, Watanabe Y, Kumagai Y, et al. Reactive cysteine persulfides and Polythiolation regulate oxidative stress and redox signaling. Proc Natl Acad Sci U S A*.* 2014;111:7606-7611.

7. Cheadle C, Vawter MP, Freed WJ, Becker KG. Analysis of microarray data using Z score transformation. J Mol Diagn*.* 2003;5:73-81.
